# The Role of Gender in Association between Emotional Intelligence and Self-Control among University Student-Athletes

**DOI:** 10.3390/ijerph182211819

**Published:** 2021-11-11

**Authors:** Audrone Dumciene, Saule Sipaviciene

**Affiliations:** 1 Department of Physical and Social Education, Lithuanian Sports University, 44221 Kaunas, Lithuania; 2 Department of Health Promotion and Rehabilitation, Lithuanian Sports University, 44221 Kaunas, Lithuania; saule.sipaviciene@lsu.lt

**Keywords:** emotional intelligence, self-control, gender, student-athletes

## Abstract

The purpose of this study was to reveal the peculiarities of undergraduate studies university student-athletes’ emotional intelligence and self-control indicators, and the role of gender as a predictor in the association between emotional intelligence and self-control. The study included students regularly involved in training at least three times a week. The sample consisted of 1395 student athletes from Lithuanian universities, among them 59.2% female and 40.8% male. For measurement, the SSRI inventory and a self-control scale were used. All values of emotional intelligence indicators were significantly higher for males than females. Estimates of the components of the self-control construct varied. The score for the healthy habits component was significantly higher for women than for men, the self-discipline component did not differ significantly, and the other three components were higher for males. Estimates of the components of the self-control construct varied. Models for predicting the values of self-control components were proposed. Only one component of the emotional intelligence construct, optimism, was repeated in all forecasting models, as well as gender. Other components of emotional intelligence vary in models.

## 1. Introduction

The expression of emotional intelligence and self-control in various human activities, including sports, has been extensively studied in recent decades. Research on emotional intelligence and self-control in students, young people, and top athletes are quite extensive [1,2,3,4,5,6,7,8]. However, researchers pay less attention to those, who train regularly three-to-four times a week only for the need for physical activity, but not for the pursuit of sporting results [9,10].

Emotional intelligence. There is no consensus among researchers on a unified concept of emotional intelligence. Three emotional intelligence models are used most often in emotional intelligence studies: ability-based models [11,12], traits-based emotional intelligence models [13], and mixed models [14]. The concept of emotional intelligence by Mayer et al. is submitted as follows, “the ability to perceive accurately, appraise, and express emotion; the ability to access and/or generate feelings when they facilitate thought; the ability to understand emotion and emotional knowledge; and the ability to regulate emotions to promote emotional and intellectual growth” [15] (p. 511). They proposed a the four-branch model “(a) accurately perceiving emotion, (b) using emotions to facilitate thought, (c) understanding emotion, and (d) managing emotion” [15] (p. 513). 

The theoretical justification of trait emotional intelligence is presented in the study [16]. The trait emotional intelligence concept model consists of five components: “adaptability, assertiveness, emotional appraisal (self and others), emotional expression, and emotion management (others)” [16] (p. 428). The trait emotional intelligence model reflects behavioral attitudes and self-perceptions related to the ability to recognize, assimilate, and use information about emotions. The trait emotional intelligence model is considered to be useful in research related to the social context [17]. The trait emotional intelligence questionnaire is mentioned as one of the best instruments for trait emotional intelligence measurements [17]. In terms of measurement, according to the review study, the traits emotional intelligence questionnaire can be considered a “gold standard” for a full-scale estimation of trait emotional intelligence [18]. The estimates of traits emotional intelligence could become important indicators of personal development in the educational and social fields, as well as in professional development [8]. Although, there are opinions [19] that the trait model better reflects emotional intelligence, [12] substantiate the completeness and suitability of the competency abilities model for emotional intelligence studies.

There are opinions that the trait model better reflects emotional intelligence than the abilities model [19]. However, the proponents of the abilities model substantiate the completeness and suitability of the competency abilities model for emotional intelligence studies [12].

Their improved emotional intelligence construct includes four emotion management abilities perceiving emotions, facilitating thought using emotion, using emotion, understanding emotions, and managing emotions [12]. Emotional intelligence reveals the ability to adapt, select, and change situations by recognizing and managing emotions and more successful athletic performance athletes engaged in a variety of sports [20,21].

Emotional intelligence is associated with satisfaction in achievement in sports [22]. The level of emotional intelligence has a potentially positive effect on the achievements of athletes and the quality of coaching activities, according to the findings in research [23]. The prognostic importance of emotional intelligence is especially relevant in self-control in competitive sports [24]. Emotional intelligence is also associated with self-control skills and the motivation of athletes themselves to play sports and achieve good results [25]. 

Although, there are studies in various fields where no significant differences were found in the values of emotional intelligence indicators in terms of gender [26,27]. The results of studies showed that training can significantly improve students’ emotional intelligence [28,29,30].

Unfortunately, the trait emotional intelligence questionnaire has not been translated, tested, and validated with Lithuanian-speaking subjects. Therefore, the Schutte self-report inventory (SSRI), based on the original Salovey and Mayer model [21], was used for this study because it has been tested and validated with a sample of Lithuanian-speaking participants [22].

Self-control. Tangney, Baumeister, and Boone define man’s ability to control himself as “arguably one of the most powerful and beneficial adaptations of the human psyche” [31] (p. 272). People are happier and healthier when they better adapt to the environment. One of the most important features of adapting to the environment is the trait of self-control [31]. Some people, thanks to self-control, can manage much better than others in their paths in life. They can better deliver on promises and achieve better results at work. Better self-control skills could likely be associated with higher achievement in certain areas of activity [31].

The concept of self-control [31] is the ability to ignore or control one’s internal reactions, to change unwanted behaviors or, in other words, the ability to control oneself [31]. Thus, in this respect, self-control is likely to have a positive effect on various human achievements in life. 

Terms that are often used in the literature as alternatives to the term self-control are self-regulation, self-discipline, and willpower [32]. However, they are not used identically. Baumeister et al. [33] argue that self-control is a specific form of self-regulation where the individual consciously and consciously seeks to control himself, and self-regulation is a more general concept that comprises automatic and involuntary regulatory processes.

Studies have shown that a feature of self-control can vary from person to person, and that may interact with environmental variables and change significantly as internal resources change [34,35]. Research has revealed the importance of self-control as a significant psychological parameter in solving problems such as unethical or immoral behavior [36] and risk-taking [37]. Decreased ego can lead to risky behaviors [37]. 

According to the proponents of the willpower concept, willpower is certain energy whose resource is specific to individuals [32,38]. Decreases in willpower energy can disrupt self-control and worsen an individual’s attention or concentration. There is no consensus on the use of willpower energy resources. There are two approaches, one is that in the process of self-control energy resources are reduced and the second is that willpower energy resources are unlimited. Researchers have revealed that individuals can be divided into two main groups: some believe that willpower energy resources are unlimited, and others that willpower energy resources decrease during self-control processes and this may be related to individual performance [39]. The results of a study showed that those who believe in the inexhaustibility of willpower energy resources achieve better performance [39]. Those who believed that in the process of self-control willpower energy resources were depleted, performed worse than the first group of subjects. The differences in the research performance of the two groups can be explained by different approaches to resources from a psychological perspective.

An ability of self-control is significant in a variety of sports and for exercise behaviors as well as athletic outcomes [40]. A review analytical study indicates that individuals with a high level of self-control manage their emotions better than individuals with a low level of self-control, achieving higher results in exercise [41]. In addition, it was revealed that various types of exercise potentially affect an individual’s ability to self-control [41]. 

It is considered that very good self-control skills are likely to be important for individuals’ physical activity [41]. The results of the study revealed that to young athletes, there are links between self-control achievement in sports competitions, motivation, and the weakening of self-control [42]. Harmful prior efforts to strengthen self-control have been identified as this may reduce athletic performance [43]. 

Different studies reveal contradictory self-control results. Females have been seen above their self-control skills than their male counterparts [44]. There are also conflicting results that male young people showed better self-control skills than females [45]. Additionally, a study in which the 16–19-year-olds displayed no significant differences in self-control indicators in terms of gender [46]. The self-control level of preschool student females was higher than pre-school student males [47]. Other researchers have noted the worse self-control level in males compared to females [48]. For top-level performance athletes, the highest level of self-control is very important and it is not clear why athletes of both genders sometimes make mistakes under pressure due to the diverse factors on the lack of self-control [49]. Scientists are increasingly expressing the view that the fluctuations of the performances of athletes are potentially determined by the decrease in self-control [50]. The contradictory results in studies may be obtained due to differences in the sample properties, environment, and research instruments.

The above overview of emotional intelligence and achievement in sport, and that athletes’ performance may be affected by self-control abilities, and thus interfaces of emotional intelligence with self-control, suggests possible reliable correlations between emotional intelligence and self-control in young athletes, and more specifically for student-athletes. Therefore, the purpose of this study was to reveal the peculiarities of undergraduate studies university student-athletes’ emotional intelligence and self-control indicators, and the role of gender as a predictor in the association between emotional intelligence and self-control.

The hypothesis is as follows: there are significant differences in emotional intelligence and self-control among university student-athletes in terms of gender, and gender is a significant predictor in the association between emotional intelligence and self-control.

## 2. Materials and Methods

### 2.1. Participants

The study was conducted at 7 of 13 state universities with over 5000 student-athletes.

The study included students regularly involved in training at least three times a week. In this study, they are called student-athletes. They are considered to be a participant in organized competitive sports, supported by the university in which the student is enrolled, but does not represent a university or national team. Participants were selected by purposive sampling. The sample consisted of 1395 undergraduate studies university full-time student-athletes from Lithuanian universities, among them 826 females and 569 males. The age of the subjects was 23.66 ± 2.23 years. Everyone participated in the study voluntarily, with no financial incentive, and they were informed of their right to terminate their participation in this investigation at any time. A purposive sampling method was used to select subjects. The research was conducted following the principles of reliability, honesty, respect, and accountability. The Ethics Committee of Social Sciences Research of the Lithuanian Sports University has issued a permit to conduct this research as meeting the ethical and legal requirements in Lithuania, where the research was conducted. The researchers provided participants with information about the study, its goals and objectives, and the progress of the study. Subjects were informed that their personal data would be processed and stored following the requirements of the Personal Data Protection Code. Subjects were provided with questionnaires, which they completed during the sessions and the duration of the process was not limited. Subjects were able to express their agreement or refusal to participate in the study by completing the questionnaire and marking one of the possible answers at the beginning of the questionnaire in the sociodemographic part of the questionnaire: “I agree to participate” or “I disagree to participate”. 

### 2.2. Instruments

The questionnaire for this investigation was based on the inventory and scale from scientific literature [31,51]. It included information on the age, gender, number of training sessions per week, emotional intelligence, and self-control indicators. The study used the two following inventory and scales: Schutte Self-Report Inventory (SSRI) [51] and the Tangney Short Self-Control Scale [31]. 

It was previously mentioned that the trait model better reflects emotional intelligence [19]. Unfortunately, the trait emotional intelligence questionnaire has not been translated, tested, and validated with Lithuanian-speaking subjects. Therefore, the Schutte Self-Report Inventory (SSRI), based on the original Salovey and Mayer model [52], was used for this study because it has been tested and validated with a sample of Lithuanian-speaking participants, and a reliability value of 0.84 was obtained for the entire SSRI [53]. The Schutte Self-Report Inventory is based on the four-branch emotional intelligence ability model [52]. The model contains four components of the emotional intelligence construct, namely optimism, social skills, appraisal, and utilization [12]. The Schutte Self-Report Inventory is most commonly used to examine emotional intelligence in terms of abilities, as noted in [17]. All of the 33 items of The Schutte Self-Report Inventory were evaluated on a Likert five-point scale, respectively: 1 = strongly disagree, 2 = disagree, 3 = neutral, 4 = agree and 5 = strongly agree. 

The internal consistency of the SSRI for this study was verified by calculating Cronbach’s alpha coefficients of subscales (Table 1) and for the whole SSRI—0.789.

The self-control scale was used in this study to evaluate the self-control level of subjects [31]. The self-control scale contains five components, namely self-discipline, non-impulsive action, healthy habits, work ethic, and reliability [31]. The self-control scale consists of 36 items that the participants were evaluated on a Likert five-point scale, namely 1 = strongly disagree, 2 = disagree, 3 = neutral, 4 = agree, and 5 = strongly agree. 

The self-control scale was translated into Lithuanian and tested with Lithuanian-speaking subjects [54]. The internal consistency of the Self-Report Inventory for this study was verified by calculating Cronbach’s alpha coefficients of subscales (Table 1) and for the whole scale—0.786.

The questionnaire was provided to the subjects in printed form and administered by the investigators. Assistance in distributing and compiling the questionnaires was provided by the coaches.

### 2.3. Statistical Analysis

Data were analyzed using descriptive statistics and IBM Statistics for Windows 22.0. The coefficients of asymmetry and excess were calculated to check the initial data distribution. As the calculated values of the coefficients ranged from −2 to 2, it can be assumed that the raw data distribution is slightly away from the nominal distribution [55]. The Student *t*-test was used to assess the difference between the mean estimates of the components of emotional intelligence and self-control constructs. Pearson correlations (two-sided) were calculated as continuous variables. Since no one correlation coefficient overshoots 0.70, the multicollinearity assumption was satisfied. A stepwise multiple regression analysis was carried out, to respond to whether and to what extent emotional intelligence indicators and gender the gender of the subjects allows you to predict self-control indicators’ values.

## 3. Results

The Cronbach alpha coefficients, means, standard deviation of the variables of emotional intelligence and self-control scales from this study results are presented in Table 1.

This study revealed that values of all components of the construct of emotional intelligence in terms of gender differ statistically significantly (*p* < 0.05). In addition, males’ self-ratings are higher than females’ in all components of the emotional intelligence construct. 

Females rated themselves better than males only on the healthy habits component of the self-control construct. Estimates of other components are higher in males than in the female. In addition, they differ significantly (*p* < 0.05). Only the estimates of the self-discipline component of the self-control construct for females and males did no differed significantly (*p* > 0.05). 

The Pearson correlation coefficients between estimates of emotional intelligence components, self-control components, and gender are shown in Table 2.

All Pearson correlation coefficients are statistically significant. These components of the emotional intelligence construct are most related to the components of the self-control construct. Optimism is most strongly correlated (r = 0.219) with non-impulsive action, social skills with non-impulsive action (r = 0.152), appraisal with reliability (r = 0.129), utilization with work ethics (r = 0.108). The gender correlates strongly with social skills (r = 0.351) and weakest with healthy habits (r = 0.079).

A stepwise multiple regression analysis was carried out, to respond to whether and to what extent emotional intelligence indicators and gender allows you to predict self-control indicators’ values. The results of linear stepwise regression analysis are shown in Table 3.

Only two study variables, the gender, and emotional intelligence component optimism, and the constant, as shown by the results of the stepwise regression analysis, are included in all five prediction models. Mostly, three components of emotional intelligence are included in the self-control components as healthy habits and reliability prediction models.

## 4. Discussions

This study was designed to test the hypothesis that there are significant gender differences in emotional intelligence and self-control among university student-athletes, and gender is a significant factor in the links between emotional intelligence and self-control.

Based on the results of the statistical analysis of the data, it can be stated that many of the assumptions raised in the hypothesis were confirmed. Estimates of the components of emotional intelligence and self-control constructs differ significantly, except for one component of self-discipline.

The university student-athlete male self-rated higher than the student-athlete female in components of emotional intelligence that optimism, social skills, appraisal, and utilization, also in components of self-control construct, non-impulsive action, work ethics, and reliability. Females, meanwhile, rated themselves significantly higher on only one component of healthy habits. Self-discipline is likely a more common characteristic of student-athletes and the differences between women and men estimates are very small (*p* > 0.05).

Our results were different in some aspects from the results of other studies. The results of our research are not contrary to the results of some other investigators, they have identified significant differences in the three emotional intelligence construct components of gender [56]. So, according to the results, although some differences in the components of emotional intelligence constructs were found in males and females, the overall assessment did not differ significantly in terms of gender [57].

There are several studies whose results did not show significant differences in emotional intelligence either as a whole or in the components of the construct in terms of gender [26,27,58]. The findings of this study confirm the results of other studies that significant differences in the components of emotional intelligence between females and males are possible [59,60,61].

Assessing the components of the self-control construct values, identified in our study, from a gender perspective, the male self-assessment scores were higher (*p* < 0.05) than the female components for non-impulsive action, work ethics, and reliability. Meanwhile, females rated themselves higher than males (*p* < 0.05) in only the component healthy habits. No difference (*p* > 0.05) was found for the component self-discipline between estimates for females and males. In our previous research, no significant gender differences of athletic and non-athletic postgraduate students were found, but males rated self-discipline and reliability higher and females rated non-impulsive actions higher [62].

The results obtained in our study are partially inconsistent with those obtained by other researchers. The university student female in some aspects has better self-control than male. However, neither gender nor self-control traits have not significantly affected the choice of exercise [62]. Highly high self-control levels can help young people stay in the chosen sport [63]. Higher, though statistically insignificant, self-control levels for females than males were determined by a representative sample of persons aged from 12 to 34 years [64].

As revealed, self-control gender differences may occur at short intervals, while at long-term intervals, these differences are insignificant because the traits of the self-control of males and females are developed by similar models. Similar relationships between self-control and social factors are characteristic of both genders also [65,66]. 

Estimates of male self-control components higher than a female can be interpreted based on the expected value of control theory [67]. Males likely expect better achievements and better rewards for these achievements.

At the time of sporting activities to achieve better results, a higher level of self-control is likely required. In this case, the self-control efforts, in other words, the depleted willpower resources, can reduce the effectiveness of another area of activity, such as the academic activity performance [41]. This reveals that persons with higher self-control levels are happier and their relationships are better [68].

Researchers pointed out that self-control skills are vital for a person, high self-control skills enable an individual to achieve more in professional or academic activities and to feel better with their well-being [68]. However, wasting a lot of effort on self-control can start to make you feel tired or weaken your motivation for the activity. These thoughts evoke the opinions of researchers who believe that the resources of self-control (willpower) are limited and dwindling [39].

Our study revealed significant Pearson correlations between all components of emotional intelligence construct components and self-control constructs components and gender. Thus, the gender factor in this study plays an important role in describing individuals’ emotional intelligence, self-control, and interrelationships.

All components of the emotional intelligence construct are positively related to the elements of the self-control construct self-discipline, non-impulsive action, healthy habits, and work ethics. Except for the component reliability, which was negatively associated with optimism, social skills, and appraisal, but positively with utilization. In this study, we found that all self-control construct components are positively associated with gender. 

Our results do not contradict the statements of other authors about the links between self-control and various human characteristics [69].

The results of the linear stepwise regression analysis revealed that all the prediction models of the self-control construct components include emotional intelligence component optimism and gender. The utilization component is one of the predictive components in models for predicting self-discipline, work ethics, and reliability components. None of the prognostic models contain all the components of the emotional intelligence construct. 

The results we obtained differed in some cases from those of other investigators, presumably due to the specific sample of subjects [2,5,7,8,21,43,54,56,57,64]. Student athletes do not represent all young people of a similar age. On one hand, this is a limitation of the study, but on the other hand, it allows a deeper study of the sample of interest to the researchers. The proposed prediction models can be useful for coaches and other sports professionals. Coaches knowing the indicators of an emotional intelligence can provisionally predict the self-control features of their trained youngsters. With data on emotional intelligence, health behavior among university students can be predicted [70]. There are also the new methodologies for predicting promising athletes using an individual characteristics and a Bayesian analysis [9] and predicting using models based on machine learning algorithms [71].

Based on the results obtained in the study, it can be stated that the statements made in the hypothesis partially were confirmed, except for the case of the self-discipline component of the self-control construct, for which the estimates of female and male did not differ significantly.

Future research would be interesting to reveal how belief in the unlimitedness of willpower resources, and the belief that willpower resources are depleted through self-control, affect athletes’ performance in sports.

### Limitations and Strengths

Several limitations of the study should be noted. The study was performed using self-report questionnaires, so the responses could be affected by the social environment. Data analysis was based on correlations and linear stepwise regression, which makes it difficult to formulate conclusions about causes and effects. The research was conducted out using self-report instrumentations and could, therefore, be influenced by the psychosocial environment of the participants. The study sample consisted of university student-athletes, but not young people of the same age as the subjects.

The advantage is that it extends the results of previous research related to emotional intelligence and self-control research. Both areas are explored quite extensively, but emotional intelligence and self-control in the common space are explored much less frequently. The novelty of this study is that it examined the association among emotional intelligence, self-control, and gender in a sample of university student-athletes. The study found significant correlations among emotional intelligence and self-control, and gender and that gender predicts the estimates of the components of the self-control construct in the student-athlete sample.

## 5. Conclusions

Significant differences were revealed in female and male university-student athlete assessments of the components of the emotional intelligence and self-control constructs, except for the self-control construct component self-discipline.

All components of emotional intelligence and self-control constructs with each other and with gender in the university student-athlete sample are significantly related. Gender is one of the most important predictors of the components of the self-control construct. The component optimism was included in all proposed prognostic models as only one of the components of the emotional intelligence construct. Each of the components of the emotional intelligence construct ‘was included in at least one of the prognostic models.

## Figures and Tables

**Table 1:** Cronbach alpha, means, and standard deviation of the variables of the total sample and by gender, and Student *t*-test.

Component of Construct	Cronbach Alpha	Total Sample *n* = 1395		Female (*n* = 826)		Male (*n* = 569)		*t*
		M	SD	M	SD	M	SD	
Schutte Self-Report Inventory								
Optimism	0.903	4.08	0.828	3.94	0.811	4.27	0.815	−7.40 ***
Social Skills	0.867	4.15	0.778	3.92	0.751	4.47	0.694	−13.97 ***
Appraisal	0.638	4.18	0.711	3.99	0.727	4.46	0.581	−13.00 ***
Utilization	0.813	3.97	0.694	3.88	0.668	4.09	0.710	−5.83 ***
Self-Control Scale								
Self-Discipline	0.721	2.98	0.421	2.97	0.434	3.00	0.402	−0.923
Non-Impulsive Action	0.664	2.96	0.446	2.94	0.449	3.07	0.538	−4.67 ***
Healthy Habits	0.784	3.03	0.663	3.08	0.663	2.97	0.657	2.94 **
Work Ethic	0.663	3.00	0.610	2.98	0.608	3.12	0.632	−4.37 ***
Reliability	0.751	3.07	0.648	2.87	0.482	3.26	0.663	−2.17 *

Notes. * *p* < 0.05 (two-tailed); ** *p* < 0.01 (two-tailed); *** *p* < 0.001 (two-tailed).

**Table 2:** Pearson correlations coefficients amongst emotional intelligence, self-control components, and gender.

Variable	1	2	3	4	5	6	7	8	9	10
Self-Discipline	1	0.145 **	0.125 **	−0.110 **	−0.303 **	0.143 **	0.054 *	0.067 *	0.091 **	0.125 **
Non-Impulsive Action	0.145 **	1	−0.108 **	0.064 *	−0.203 **	0.219 **	0.152 **	0.072 *	0.083 **	0.124 **
Healthy Habits	0.125 **	−0.108 **	1	−0.122 **	0.098 **	0.131 **	0.115 **	0.108 **	0.081 *	0.079 **
Work Ethics	−0.110 **	0.064 *	−0.122 **	1	−0.111 **	0.122 **	0.076 *	0.086 *	0.122 **	0.116 **
Reliability	−0.303 **	−0.203 **	0.098 **	−0.111 **	1	−0.130 **	−0.089 **	−0.129 **	0.108 **	0.058 *
Optimism	0.143 **	0.219 **	0.131 **	0.122 **	−0.130 **	1	0.226 **	0.226 **	0.170 **	0.194 **
Social Skills	0.054 *	0.152 **	0.115 **	0.076 *	−0.089 **	0.226 **	1	0.357 **	0.308 **	0.351 **
Appraisal	0.067 *	0.072 *	0.108 **	0.086 *	0.129 **	0.226 **	0.357 **	1	0.252 **	0.329 **
Utilization	0.091 **	0.083 **	0.081 *	0.122 **	0.108 **	0.170 **	0.308 **	0.252 **	1	0.154 **
Gender	0.125 **	0.124 **	0.079 **	0.116 **	−0.058 *	0.194 **	0.351 **	0.329 **	0.154 **	1

Notes. * *p* < 0.05 (two-tailed); ** *p* < 0.01 (two-tailed). 1—self-discipline, 2—non-impulsive action, 3—healthy habits, 4—work ethics, 5—reliability, 6—optimism, 7—social skills, 8—appraisal, 9—utilization, 10—gender.

**Table 3:** The results of linear stepwise regression analysis of study variables.

Model	*R*	*R* ^2^	*R*^2^ Adjusted	*R*^2^ Change	*F* (*df*)	*β*	*β* Standardized	*t*
	Dependent variables: components of the self-control scale							
	Independent variables: components of the emotional intelligence inventory and gender							
Dependent variable: self-discipline								
Model 1	0.156	0.024	0.023	0.018	17.685 (1393) **			
Constant						2.824		12.89 ***
Optimism						0.328	0.287	5.341 ***
Utilization						0.321	0.305	1.157 *
Gender						0.218	0.197	2.458 **
Dependent variable: non-impulsive action								
Model 2	0.364	0.167	0.143	0.101	9.963 (1393) **			
Constant						3.237		15.55 ***
Optimism						−0.238	−0.220	−3.461 ***
Social Skills						0.438	0.418	2.562 **
Gender						0.192	0.188	2.511 **
Dependent variable: healthy habits								
Model 3	0.363	0.132	0.11	0.058	10.586 (1392) **			
Constant						2.916		12.833 ***
Optimism						0.528	0.363	3.096 **
Social Skills						0.427	0.401	3.051 **
Appraisal						−0.125	−0.108	2.842 **
Gender						0.452	0.415	2.088 *
Dependent variable: work ethics								
Model 4	0.388	0.151	0.15	0.15	18.3 (1392) ***			
Constant						4.543		10.95 ***
Utilization						−0.544	−0.478	4.317 ***
Optimism						0.238	0.226	3.015 **
Gender						0.387	365	1.251 *
Dependent variable: reliability								
Model 5	0.428	0.178	0.177	0.028	8.983 (64) ***			
Constant						2.817		13.008 ***
Optimism						0.518	0.347	4.056 ***
Appraisal						0.487	0.388	2.312 *
Utilization						0.258	0.225	2.587 **
Gender						0.359	0.312	2.098 *

Notes. * *p* < 0.05 (two-tailed); ** *p* < 0.01 (two-tailed); *** *p* < 0.001 (two-tailed).

## Data Availability

The data used to support the findings of this study are available from the corresponding author upon request.
